# Pheromone Race Composition of *Ostrinia nubilalis* (Lepidoptera: Crambidae) and Larval Co-Occurrence with *Sesamia nonagrioides* (Lepidoptera: Noctuidae) in Maize in Central-Eastern Italy

**DOI:** 10.3390/insects16121267

**Published:** 2025-12-13

**Authors:** Maria Chiara Battistelli, Diego Palpacelli, Giorgio Sperandio, Matteo Pacella, Fabio Ramilli, Sara Ruschioni, Abdalhadi M. A. Abulebda, Paola Riolo

**Affiliations:** 1 Department of Agricultural, Food and Environmental Sciences, Polytechnic University of Marche, Via Brecce Bianche, 60131 Ancona, Italy; m.c.battistelli@pm.univpm.it (M.C.B.); g.sperandio@staff.univpm.it (G.S.); m.pacella@staff.univpm.it (M.P.); a.abulebda@pm.univpm.it (A.M.A.A.); p.riolo@staff.univpm.it (P.R.); 2 Instituto de Biología Molecular y Celular de Plantas (Consejo Superior de Investigaciones Científicas-Universitat Politècnica de Valencia), 46022 Valencia, Spain; dpalpac@doctor.upv.es; 3 Department of Pharmacy and Biotechnology (FaBiT), University of Bologna, Via S. Donato, 15, 40127 Bologna, Italy; fabio.ramilli2@unibo.it

**Keywords:** *Ostrinia nubilalis*, *Sesamia nonagrioides*, corn borers, species co-occurrence, monitoring

## Abstract

*Ostrinia nubilalis* (Lepidoptera: Crambidae) and *Sesamia nonagrioides* (Lepidoptera: Noctuidae) are major maize pests in the central-eastern Mediterranean area, responsible for both quantitative and qualitative yield losses. This study investigated the corn borer species present in central-eastern Italy, the presence and prevalence of *O. nubilalis* pheromone races, and the within-plant larval distribution. Traps baited with E, Z, or E/Z pheromone lures were used to investigate *O. nubilalis* pheromone races, while 596 maize plants were sampled at harvest and dissected to determine species composition, relative abundance, and larval distribution across three plant sections (lower and upper stalk and ear). Males were captured in traps baited with each pheromone lure, with no significant differences among catches. Larval sampling revealed the co-occurrence of *O. nubilalis* and *S. nonagrioides*, with *O. nubilalis* being the prevalent species. For both species, larvae were significantly more abundant in the lower plant section, and co-occurrence did not appear to influence their within-plant distribution. Comparing the species-specific larval distribution, a significantly higher proportion of *S. nonagrioides* occurred in the lower plant section, whereas *O. nubilalis* was significantly more frequent in the ears. However, the presence of both species in the ears highlights the need to consider both pests in management. These findings highlight the importance of monitoring activities in the area, using traps baited with each pheromone lure for *O. nubilalis*. Knowledge of the co-occurrence between the two species can be considered fundamental for developing Integrated Pest Management strategies, as it can influence the timing and effectiveness of control methods.

## Simple Summary

*Ostrinia nubilalis* and *Sesamia nonagrioides* are important maize pests in central-eastern Mediterranean Europe, with larvae boring into plants, causing yield losses. This study investigated the corn borer species present in central-eastern Italy, the presence and prevalence of *O. nubilalis* pheromone races, and the within-plant larval distribution across three plant sections (lower and upper stalk and ear). Traps baited with E, Z or E/Z pheromone lures captured *O. nubilalis* adults, with no significant differences among lures. Larval sampling revealed the prevalence of *O. nubilalis* over *S. nonagrioides*, a higher occurrence of both species in the lower plant section, and the presence of both species in the ears. Co-occurrence of the two species within the same plant did not affect species-specific larval distribution. These findings provide useful information on corn borers in central-eastern Italy and highlight the importance of monitoring activities for their management, particularly when multiple species may co-occur.

## 1. Introduction

The European corn borer (ECB), *Ostrinia nubilalis* (Hübner, 1796) (Lepidoptera: Crambidae), and the Mediterranean corn borer (MCB), *Sesamia nonagrioides* (Lefèbvre, 1827) (Lepidoptera: Noctuidae), are considered two of the most important maize pests in central-eastern Mediterranean Europe [1,2,3].

Damage to maize results from the feeding activity of the corn borer larvae, which exhibit cryptic behavior and bore into maize plants until pupation [4,5]. The typical tunneling activity of corn borers causes both quantitative and qualitative losses [4,6], reducing yield and facilitating the entry of fungal pathogens, including mycotoxin-producing species (mainly *Fusarium* spp. and *Aspergillus* spp.) [4,7,8]. Mycotoxins are currently considered one of the major challenges in maize production, as their accumulation in the ears can compromise grain use for both animal and human consumption due to associated safety risks [7].

*Ostrinia nubilalis* is a cosmopolitan and polyphagous species, native to Europe and now established in Africa, North America, and Eurasia [8,9]. In Italy, the species has been reported from several regions, particularly in the central and northern areas (including Veneto, Piedmont, Lombardy, Emilia-Romagna, Marche, Tuscany, and Campania) [10,11,12,13,14,15]. The ECB exhibits sex pheromone polymorphism, with two races (E and Z) that differ in pheromone composition and mating behavior [9,12]. Females of different races produce, and males respond, to distinct blends of the pheromone components (E)- and (Z)-11-tetradecenyl acetate (96:4 to 99:1 E:Z ratio for the E race and 3:97 E:Z ratio for the Z race), promoting assortative mating [9,16,17,18]. Where both races occur in sympatry, inter-race mating can occur [14,15], resulting in hybrids that primarily respond to an intermediate 65:35 E:Z blend [12,19]. Both the E and Z races of *O. nubilalis* have been documented across the Italian peninsula [13,14,15,20], with a prevalence of the E race in Lombardy [13] and Emilia-Romagna [12,15], and of the Z race in Campania [15]. Hybrid individuals have also been reported in regions where both races occur in sympatry, such as Emilia-Romagna [14,15], Tuscany and Campania [15]. However, available data on race distribution remain geographically limited and, in some cases, outdated. Pheromone-baited traps are considered one of the most cost-effective approaches to detect adult emergence and flight peaks of corn borer species and, consequently, to determine the optimal timing for control measures [21]. To optimize the pheromone-based monitoring strategy for ECB, the locally dominant pheromone race should be identified beforehand [4]. *Ostrinia nubilalis* overwinters as diapausing fifth-instar larvae, mainly sheltered within the lower part of the maize stalk, between the soil surface and the ear [22,23,24]. Pupation and adult emergence usually occur in early spring [8], and two ECB biotypes are recognized based on voltinism: a univoltine biotype (frequent above 46° N) and a multivoltine biotype [6]. The multivoltine biotype is common in northern Italy, where two generations per year are usually recorded [6], and a partial third generation may occur under favorable environmental conditions [13,20].

*Sesamia nonagrioides* is an oligophagous species primarily feeding on Poaceae, with maize as the major host in the Mediterranean area [25]. Its distribution ranges from approximately 31° N to 46° N latitude, including countries such as Italy, Spain and Portugal [26]. As in the case of *O. nubilalis*, *S. nonagrioides* overwinters as last-instar larvae in maize stalks and residues [3,27], and it is characterized by lower cold tolerance compared to the former species [28]. In Europe, the MCB usually completes two to four generations per year, depending on environmental conditions [26]. In regions with sufficiently high temperatures and continuous availability of suitable host plants, the MCB can remain active throughout the year, although activity may be reduced during colder periods [29]. In Italy, *S. nonagrioides* has been primarily reported in the central and southern regions (Lazio, Apulia, Marche, Sicily and Sardinia), feeding on maize and other potential host plants (e.g., *Gladiolus* sp., *Strelitzia reginae*, *Trachycarpus fortunei*, *Washingtonia filiform*) [11,29,30,31,32,33,34,35].

Management of these pests is challenging, as the cryptic behavior of the larvae limits the possibility of reaching them with treatments [4,36,37]. Consequently, precise timing is essential, targeting the period of egg laying and hatching when larvae feed on leaf surfaces before entering the stalks [38]. In addition to timing, the efficacy of control methods may vary depending on the target species. For example, the egg parasitoid widely used for ECB control, *Trichogramma brassicae*, has shown limited effectiveness against *Sesamia* spp., likely due to differences in oviposition sites (i.e., *O. nubilalis* eggs on leaf surfaces versus *Sesamia* spp. eggs between leaf sheets and stalks or cobs) [8,32,37].

Given the overlapping distribution of the two species in the Italian peninsula, their co-occurrence in maize fields is possible. Co-occurrence of multiple species can further complicate corn borer management, as the biological cycles of different species must be considered when determining control timing and methods.

Studying the presence, abundance, and potential interactions among corn borer species sharing the same area and host plant is therefore crucial for implementing Integrated Pest Management (IPM) strategies, as it helps determine whether, when, and which control measures can be used. Species-specific ecological traits, such as the sex pheromone polymorphism of *O. nubilalis* or the within-plant distribution of corn borer larvae, should be taken into account, as they can help optimize monitoring and field management [4,27,39]. The Marche region represents a particularly interesting area for investigating these aspects, as both corn borer species have been reported [11,40], although never co-occurring on maize.

In this study, we aimed to: (i) identify the corn borer species present in the coastal area of Marche region (central-eastern Italy); (ii) assess the presence and prevalence of *O. nubilalis* pheromone races using sex pheromone baited traps to provide supplementary information on species composition; and (iii) investigate the abundance and distribution of corn borer larvae within plants in cases of potential co-occurrence.

## 2. Materials and Methods

### 2.1. Study Area

This study was conducted in a maize field located in the coastal area of the Ancona province, central-eastern Italy (Senigallia, 43°42′08″ N; 13°11′57″ E, 7 m a.s.l.) in 2016. The field (6.5 ha), previously cultivated with wheat, was sown with grain maize (hybrid DKC6092, Monsanto Italia, Milan, Italy) on April 15 at a density of 7 plants/m^2^ and harvested on September 28. Irrigation was provided weekly from early July to mid-August using a sprinkler system, and no insecticide treatments were applied during the growing season. After harvest, crop residues were incorporated into the soil during tillage operations.

### 2.2. Adult Captures

**Figure 1:**
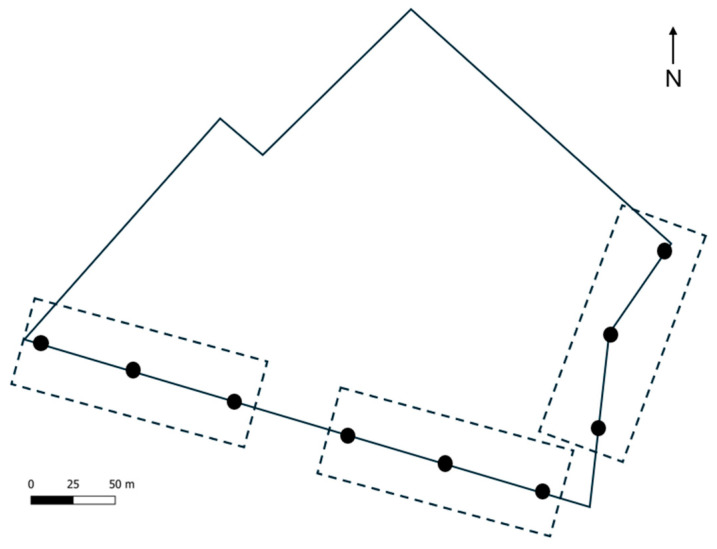
Schematic representation of trap placement within the field. Black dots represent individual cone traps, and dashed lines indicating the three experimental blocks.

Adult males of *O. nubilalis* were captured using nine cone traps (Coretrap^®^, by Riff98, Bologna, Italy) baited with E, Z or E/Z commercial pheromone lures (Isagro^®^, Milan, Italy). Traps were arranged in three blocks, each containing one trap per pheromone lure. Traps were spaced 60 m apart and positioned along the edge of the field, where vegetative borders provided aggregation sites for adults [24,41] (Figure 1). Captures were recorded and traps were emptied weekly from 12 July to 28 September. Pheromone lures were replaced every two weeks.

### 2.3. Larval Sampling

For larval sampling, the field was divided into 24 subareas of approximately equal size. Within each subarea, 25 maize plants were randomly selected at harvest, cut at ground level, and then stored under natural environmental conditions until dissection (during November). All plants were inspected for corn borer damage symptoms (e.g., stalk tunneling and entry holes) and dissected to detect and collect larvae. Plants were classified as infested when either corn borer damage symptoms or larvae were present. During dissection, both infested and non-infested plants were divided into three sections to investigate the within-plant larval distribution: the lower section (LS, below the main ear, around 90 cm above ground), the upper section (US, above the main ear), and the ears (E), following the approach adopted by Velasco et al. [39] with slight modifications. Larvae from each section were counted and identified to species level under a stereomicroscope (Leica^®^ EZ4W; Heerbrugg, Switzerland). Identification was based on morphological traits, with *O. nubilalis* distinguished by the six piliferous tubercles clearly visible on the dorsal surface of each body segment [8,24,33], and *S. nonagrioides* identified according to the chaetotaxy of the ninth urotergite, following Pollini [33].

### 2.4. Statistical Analysis

Data on male captures were used to determine the predominant pheromone race of *O. nubilalis* in the study area [13,21]. A randomized block analysis of variance (ANOVA) was used to test for differences in male trap captures among pheromone lures, including trap position (block) as a fixed factor to account for potential spatial variation (α = 0.05).

Differences in larval incidence (presence/absence) between the two corn borer species at the plant level were tested using McNemar’s test with continuity correction (α = 0.05), considering each plant as a paired observation for the two species. Paired Wilcoxon signed-rank tests were used to compare larval abundance per plant (α = 0.05).

Larval abundance of *O. nubilalis* and *S. nonagrioides* across plant sections was analyzed using a generalized linear mixed model (GLMM) with a Poisson distribution, considering all sampled plants. Species, plant section, and their interaction were included as fixed effects, while plant ID was included as a random intercept. Model fitting was performed using the glmer() function from the lme4 package [42] (version 1.1.37) and validated with DHARMa package [43] (version 0.4.7) to assess dispersion, uniformity, and outliers. Post hoc pairwise comparisons of estimated marginal means were carried out using the emmeans package [44] (version: 1.11.2), with Tukey adjustment.

To assess species-specific within-plant larval distribution patterns, only plants hosting either *O. nubilalis* or *S. nonagrioides* larvae were considered. For each species, observed larval frequencies per plant section were compared with an expected uniform distribution (i.e., one-third of larvae in each plant section) using chi-squared goodness-of-fit tests (α = 0.05).

Plants hosting a single species were also used to compare within-plant distribution between the two corn borer species, using chi-squared tests of independence (α = 0.05). When pairwise post hoc comparisons among plant sections or between species were conducted, *p*-values were adjusted using the Bonferroni correction to account for multiple testing. The same approach was applied to evaluate the effect of co-occurrence on species-specific within-plant distribution patterns, by comparing the distribution of each species when occurring alone versus in co-occurrence.

All statistical analyses were performed in R (version 4.4.1).

## 3. Results

### 3.1. Adult Captures

A total of 678 *O. nubilalis* males were captured during the trapping period: 284 individuals in E-baited traps, 289 in E/Z-baited traps and 105 in Z-baited traps. Although the total number of captures in Z-baited traps was lower than in E- and E/Z-baited traps, no significant differences in mean captures among the three pheromone lures were detected (ANOVA, F_2,4_ = 2.14, *p* = 0.23, η^2^ = 0.49). Similarly, trap position (block) had not significant effect (ANOVA, F_2,4_ = 0.55, *p* = 0.61, η^2^ = 0.22).

### 3.2. Corn Borer Larval Incidence and Abundance

Larval sampling revealed the presence of both *O. nubilalis* and *S. nonagrioides*.

Out of the 600 plants collected, 596 were dissected, while four completely broken plants were excluded from the sample. Signs of infestation were observed in 592 (99.3%) of the 596 dissected plants, and larvae were detected in 542 plants (90.9%).

A total of 1926 larvae were collected, of which 1222 (63.4%) were *O. nubilalis* and 704 (36.6%) were *S. nonagrioides*.

*Ostrinia nubilalis* was detected in 471 plants (79.0% of the total dissected), whereas *S. nonagrioides* was found in 386 plants (64.8%), with *O. nubilalis* showing a significantly higher larval incidence than *S. nonagrioides* (McNemar’s χ^2^_1_ = 31.08, *p* < 0.001). The mean number of larvae (±S.D.) per plant hosting the species was 2.6 ± 1.7 for *O. nubilalis* and 1.8 ± 1.0 for *S. nonagrioides*. A significantly higher larval abundance of *O. nubilalis* compared to *S. nonagrioides* was observed (Wilcoxon signed-rank test V = 83,073, *p* < 0.001).

### 3.3. Corn Borer Larvae Distribution

**Figure 2:**
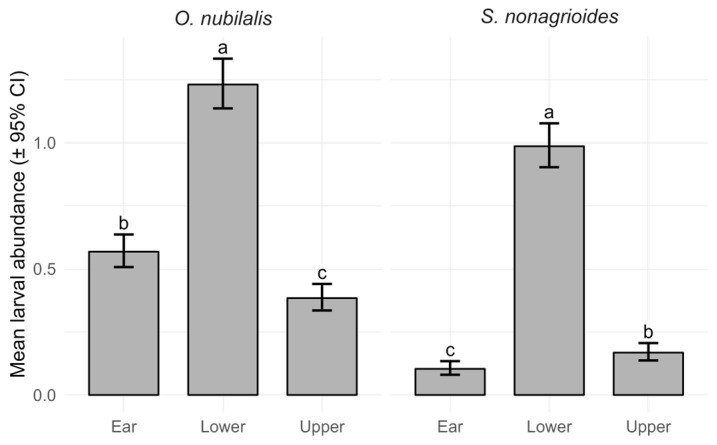
Mean larval abundance (±95% confidence interval) of *Ostrinia nubilalis* and *Sesamia nonagrioides* across plant sections (Ear, Lower, and Upper). Values represent model-predicted marginal means from a GLMM with a Poisson distribution and plant ID included as a random effect. For each species, different letters indicate statistically significant differences among plant sections based on Tukey-adjusted post hoc comparisons (*p* < 0.001).

Larval abundance varied significantly according to species (χ^2^_1_ = 142.1, *p* < 0.001), plant section (χ^2^_2_ = 280.2, *p* < 0.001), and their interaction (χ^2^_2_ = 101.5, *p* < 0.001) (Figure 2).

Specifically, *O. nubilalis* exhibited higher larval abundance than *S. nonagrioides* across all plant sections (Tukey-adjusted *p* < 0.001). Within each species, larval abundance differed significantly among plant sections (Tukey-adjusted *p* < 0.05). For *O. nubilalis*, the lower section showed the highest larval abundance, followed by the ear and the upper section (Ear–Lower: z = −11.43, *p* < 0.001; Ear–Upper: z = 4.44, *p* < 0.001; Lower–Upper: z = 14.94 *p* < 0.001). For *S. nonagrioides*, the lower section was also the most infested part, followed by the upper section and the ear (Ear–Lower: z = −16.36, *p* < 0.001; Ear–Upper: z = −2.90, *p* < 0.05; Lower–Upper: z = 15.90, *p* < 0.001). Model diagnostics indicated an adequate fit, with no significant issues in dispersion (*p* = 0.06), residual uniformity (*p* = 0.92), or outlier frequency (*p* = 0.17).

Larvae of both species were found together in 315 plants, representing 58.1% of all plants hosting larvae. Among the remaining plants, *O. nubilalis* occurred alone in 156 plants and *S. nonagrioides* in 71.

**Figure 3:**
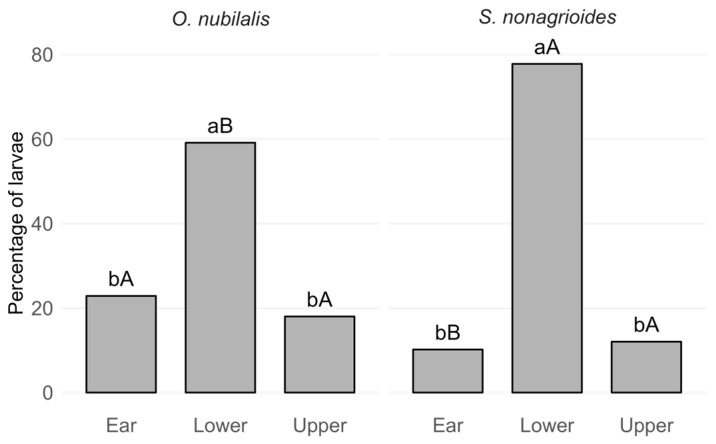
Percentage of larvae of *Ostrinia nubilalis* and *Sesamia nonagrioides* across plant sections (Ear, Lower, and Upper) in single-species plants. Lowercase letters indicate significant differences among plant sections within the same species, compared with an expected uniform distribution (one-third of larvae per section) (Chi-squared goodness-of-fit test, α = 0.05, Bonferroni-corrected for pairwise comparisons). Uppercase letters indicate significant differences between species within the same plant section (Chi-squared test of independence, α = 0.05).

In plants hosting *O. nubilalis* only, larval distribution across the three plant sections deviated significantly from a uniform distribution (Chi-squared goodness-of-fit test: χ^2^_2_ = 124.5, *p* < 0.001). Pairwise comparisons with Bonferroni correction showed that the lower section hosted significantly more larvae than both the ear and upper section (*p* < 0.001), whereas no significant difference was detected between the ear and the upper section (*p* = 0.30) (Figure 3). Similarly, in plants hosting *S. nonagrioides* only, larval distribution across the three plant sections also deviated significantly from a uniform distribution (Chi-squared goodness-of-fit test: χ^2^_2_ = 96.1, *p* < 0.001). Pairwise comparisons with Bonferroni correction indicated that the lower section hosted significantly more larvae than both the ear and the upper section (*p* < 0.001), while no significant difference was observed between the ear and the upper section (*p* = 1) (Figure 3).

In plants hosting a single species, the proportion of larvae differed between *O. nubilalis* and *S. nonagrioides* depending on the plant section. Specifically, the Chi-squared test across all sections indicated a significant overall difference (χ^2^_2_ = 13.34, *p* < 0.005).

Pairwise comparisons of proportions with continuity correction and Bonferroni adjustment (Figure 3) showed no significant difference in the upper section (χ^2^_1_ = 1.78, *p* = 0.55), whereas the lower section contained a significantly higher proportion of *S. nonagrioides* compared to *O. nubilalis* (χ^2^_1_ = 11.98, *p* < 0.005). Conversely, the ear showed a significantly lower proportion of *S. nonagrioides* compared to *O. nubilalis* (χ^2^_1_ = 7.76, *p* < 0.05).

Larval distribution across plant sections did not differ significantly for *O. nubilalis* (χ^2^_1_ = 3.25, *p* = 0.20) or *S. nonagrioides* (χ^2^_1_ = 0.76, *p* = 0.68), whether occurring alone or together with the other species (Table 1).

To further investigate the spatial distribution of *O. nubilalis* and *S. nonagrioides* in cases of co-occurrence, only plants hosting both species were considered. In 230 cases (73.0%), larvae of the two species co-occurred within at least one plant section, whereas in the remaining 85 plants (27.0%), *O. nubilalis* and *S. nonagrioides* were found totally separated into different sections. Co-occurrence was observed primarily in the lower section (211 instances), followed by the ear (23 instances) and the upper section (21 instances).

## 4. Discussion

Two corn borer species, *O. nubilalis* and *S. nonagrioides*, were identified in this study, representing the first report of their co-occurrence in maize in central-eastern Italy. *Sesamia nonagrioides* had previously been reported in the area [11], and its shift to maize-growing systems was therefore expected. However, surveys conducted between 1993 and 1996 in the coastal hills of the Marche region recorded only *O. nubilalis* and *S. cretica* on maize [40], suggesting potential differences in the spatial distribution of *Sesamia* species. Specifically, *S. cretica* seems to be common in inland areas, whereas *S. nonagrioides* seems more closely associated with the coastal area (Riolo, personal communication).

Climate warming may cause the expansion of the potential distribution area of thermophilic species such as *Sesamia* spp. [45,46], but also increase the abundance of the species in areas where they are already present, as milder winters may enhance larval overwintering survival. This may lead to an increase in co-occurrence cases among multiple corn borer species in maize fields, which could extend to additional regions and pose significant challenges for pest management.

Knowing the species composition present in a field is the first step toward effective pest management. Field inspections are therefore strongly recommended, especially in areas where multiple species may co-occur. Since infestation symptoms are similar among corn borer species, plant dissection is required to identify the species present. Practical guidelines for assessing *O. nubilalis* infestation, such as inspecting transects of 20 plants at multiple points and dissecting symptomatic plants to check for live larvae [24], provide a feasible approach to assess species composition throughout the growing season.

The detection of *S. nonagrioides* on maize in the study area highlights the importance of field inspections, as its presence can influence pest management in different ways. First, its biological cycle differs from that of *O. nubilalis* [4], potentially resulting in different egg-laying and larval exposure periods, which can affect the timing of control interventions. Since timing is crucial for effective corn borer management [4], annual monitoring of adult populations of both species is recommended to support early detection and precise scheduling of control measures. Furthermore, the typical oviposition behavior of *S. nonagrioides* can reduce the effectiveness of natural control agents commonly used against *O. nubilalis*, such as *Trichogramma brassicae* [32]. Consequently, the use of alternative egg parasitoids targeting both species (e.g., *Trichogramma evanescens*) [4,37], along with practices that support generalist predators effective against corn borers (Coccinellidae, Anthocoridae, Pentotomidae, Chrysopidae, etc.) should be considered [4,37]. Since many natural enemies inhabiting maize fields can be negatively affected by insecticide applications [37], minimizing chemical applications is the first step to preserve beneficial organisms and support IPM strategies.

Adult captures of *O. nubilalis* occurred in traps baited with E, Z, and E/Z pheromone lures, consistent with previous studies in other areas of the Italian peninsula [15]. Although Camerini et al. [13] reported significant differences in captures between E- and Z- baited traps in Pavia province, our study found no significant differences among catches from the three pheromone lures. Therefore, using traps baited with each pheromone lure is recommended in the study area to ensure timely detection and effective monitoring of adult activity. This strategy also serves as a precautionary measure against potential effects of race hybridization, which may unpredictably alter the proportions of insects responding to specific pheromone blends.

Plant dissections revealed nearly all plants were infested, either showing symptoms or hosting larvae, highlighting the importance for effective pest management to reduce crop damage [3,39]. *Ostrinia nubilalis* emerged as the prevalent species in terms of larval incidence and abundance, consistent with previous records from the Marche region [40]. However, given the potential increase in conditions favorable to *Sesamia* spp., systematic surveillance will be essential to monitor changes in species composition in the coming years.

Both species exhibited significantly higher larval abundance and proportion in the lower plant section, consistent with their known preference for the basal part of the stalk as overwintering site [22,23,39,40,47]. Downward movement provides several advantages for larval overwintering, including insulation, shelter, and reduced risk of displacement during harvesting operations [3,23,47]. *Sesamia nonagrioides* showed a higher proportion of larvae in the lower plant section than *O. nubilalis*, likely reflecting its stronger reliance on favorable microclimatic conditions at the plant base to ensure overwintering survival [3,27,46], particularly in the northern part of its distribution range. This finding may also suggest a greater contribution of *S. nonagrioides* to stalk lodging, potentially leading to higher crop losses during mechanized harvesting [48,49].

Co-occurrence of *O. nubilalis* and *S. nonagrioides* within the same plant was observed in over half of the cases and did not appear to affect species-specific larval distribution. The lower plant section remained the one hosting the highest proportion of larvae of both species and, moreover, the highest number of section-sharing events. These results provide evidence of potential coexistence among different corn borer species on maize [3,39], as previously reported in Spain (*O. nubilalis* and *S. nonagrioides*) [39] and central-eastern Italy (*O. nubilalis* and *S. cretica*) [40]. However, further studies are needed to confirm these preliminary field data and to better understand intra- and interspecific interactions in cases of niche overlap.

In the ears, *O. nubilalis* was more prevalent than *S. nonagrioides*, both in terms of larval abundance and proportion. However, the presence of both species in this plant section suggests that each can contribute to ear damage, directly through feeding and ear dropping [24] and indirectly by acting as vectors of mycotoxigenic fungi and promoting mycotoxin accumulation [7,35,50]. Consequently, effective field management should target both species to minimize yield losses and reduce contamination risk.

During mechanical harvesting of grain maize, abundant crop residues remain in the field, including stalks, roots, and cobs. Post-harvest practices, such as shredding and incorporation, increase mortality of overwintering larvae, providing a practical example of a measure that can target both *O. nubilalis* and *Sesamia* spp. [3,6,27]. These practices should ideally be implemented before spring (i.e., before March), preferably during autumn or winter (from October onwards), to combine the effects of mechanical disruption and low-temperature exposure.

A summary of key activities for corn borer management is provided in Table 2.

## 5. Conclusions

This study provides the first report of the co-occurrence of *O. nubilalis* and *S. nonagrioides* in maize in central-eastern Italy, highlighting potential implications for corn borer management. Data on species composition and *O. nubilalis* captures emphasize the importance of monitoring both species, and, for *O. nubilalis*, using traps baited with each pheromone lure to ensure timely detection and effective monitoring of adult activity.

*Ostrinia nubilalis* emerged as the prevalent species in terms of incidence and larval abundance, confirming its relevance in the surveyed area.

For the two species, larvae were most abundant in the lower plant sections, and species-specific distribution patterns remained stable whether occurring alone or together, suggesting potential for coexistence within the same host plant.

Their presence in the ears indicates potential for direct and indirect damage, emphasizing that effective management should target both species while considering their different biological characteristics.

Although limited to a single site and year, this study provides important insights into the corn borer populations in central-eastern Italy, describing species composition, the pheromone races of *O. nubilalis* based on capture data, and the within-plant larval distribution of both species under condition of co-occurrence. These findings highlight the importance of monitoring activities, including trap utilization and plant dissection, for corn borer management and provide suggestions for implementing IPM strategies in areas where multiple species co-occur. Further studies across multiple areas are needed to validate and expand these findings, and to assess the species-specific impacts on maize yield and quality.

## Figures and Tables

**Table 1:** Percentage of larvae of *Ostrinia nubilalis* and *Sesamia nonagrioides* across plant sections (LS—Lower section, US—Upper section, and E—Ear) when occurring alone (single-species) or together with the other species (co-occurrence). Larval distribution did not differ significantly between single-species and co-occurrence conditions for either *O. nubilalis* or *S. nonagrioides*, as evaluated using a Chi-squared test of independence (α = 0.05).

Species	Variable	Single-Species (%)	Co-Occurrence (%)
*O. nubilalis*	Larvae in LS	59.1	55.0
	Larvae in US	18.0	17.4
	Larvae in E	22.9	27.6
*S. nonagrioides*	Larvae in LS	77.8	78.5
	Larvae in US	12.0	13.6
	Larvae in E	10.2	7.9

**Table 2:** Summary of key corn borer management activities at different timings (pre-sowing, growing season, post-harvest), with corresponding recommendations and aims.

Timing	Type of Activity	Recommendations	Aims
Early spring/Pre-sowing—Growing season	Monitoring	Annual adult monitoring of corn borer species. For *O. nubilalis*, use pheromone traps baited with each E, Z, and E/Z pheromone lures.	Early detection and monitoring of adults to optimize timing of interventions.
Growing season	Field inspections	Sample and dissect symptomatic plants at multiple locations in the field to identify larvae.	Assess the species composition present in the field.
Growing season	Control measures	Chemical, microbiological, or biological treatments applied based on adult monitoring data and egg mass scouting (if possible).	Improve control efficacy by targeting susceptible stages (e.g., eggs and larvae before entering the stalk).
Growing season	Biological control	Select biological control agents considering the corn borer species present (e.g., in the case of egg parasitoids) and promote generalist predators.	Enhance management through complementary strategies, reducing reliance on chemical products.
Post-harvest	Agronomic control	Shred and incorporate crop residues between October and March, preferably in autumn or winter.	Increase mortality of overwintering larvae of corn borers via mechanical disruption combined with low-temperature exposure.

## Data Availability

The original contributions presented in this study are included in the article. Further inquiries can be directed to the corresponding author.

## Abbreviations

The following abbreviations are used in this manuscript:

ECB	European corn borer
MCB	Mediterranean corn borer
IPM	Integrated pest management
LS	Lower section
US	Upper section
E	Ears
GLMM	Generalized linear mixed models
